# Comprehensive genomic characterization of cotton cationic amino acid transporter genes reveals that *GhCAT10D* regulates salt tolerance

**DOI:** 10.1186/s12870-022-03829-w

**Published:** 2022-09-15

**Authors:** Xiugui Chen, Zhe Wu, Zujun Yin, Yuexin Zhang, Cun Rui, Jing Wang, Waqar Afzal Malik, Xuke Lu, Delong Wang, Junjuan Wang, Lixue Guo, Shuai Wang, Lanjie Zhao, Bobokhonova Zebinisso Qaraevna, Chao Chen, Xiuping Wang, Wuwei Ye

**Affiliations:** 1 Institute of Cotton Research of Chinese Academy of Agricultural Sciences/Zhengzhou Research Base, State Key Laboratory of Cotton Biology, School of Agricultural Sciences, Zhengzhou University, Anyang, 455000 Henan China; 2 Institute of Coastal Agriculture, Hebei Academy of Agriculture and Forestry Sciences, Tangshan, 063299 Hebei China; 3 Department of Cotton Growing, Genetics, Breeding and Seed, Tajik Agrarian University Named Shirinsho Shotemur Dushanbe, Dushanbe, Republic of Tajikistan

**Keywords:** Cationic amino acid transporter, Cotton, *GhCAT10D*, Salt stress, Gene network, Nitric oxide

## Abstract

**Background:**

The cationic amino acid transporters (CAT) play indispensable roles in maintaining metabolic functions, such as synthesis of proteins and nitric oxide (NO), biosynthesis of polyamine, and flow of amino acids, by mediating the bidirectional transport of cationic amino acids in plant cells.

**Results:**

In this study, we performed a genome-wide and comprehensive study of 79 *CAT* genes in four species of cotton. Localization of genes revealed that *CAT* genes reside on the plasma membrane. Seventy-nine *CAT* genes were grouped into 7 subfamilies by phylogenetic analysis. Structure analysis of genes showed that *CAT* genes from the same subgroup have similar genetic structure and exon number. RNA-seq and real-time PCR indicated that the expression of most *GhCAT* genes were induced by salt, drought, cold and heat stresses. *Cis*-elements analysis of *GhCAT* promoters showed that the *GhCAT* genes promoters mainly contained plant hormones responsive elements and abiotic stress elements, which indicated that *GhCAT* genes may play key roles in response to abiotic stress. Moreover, we also conducted gene interaction network of the GhCAT proteins. Silencing *GhCAT10D* expression decreased the resistance of cotton to salt stress because of a decrease in the accumulation of NO and proline.

**Conclusion:**

Our results indicated that *CAT* genes might be related with salt tolerance in cotton and lay a foundation for further study on the regulation mechanism of *CAT* genes in cationic amino acids transporting and distribution responsing to abiotic stress.

**Supplementary Information:**

The online version contains supplementary material available at 10.1186/s12870-022-03829-w.

## Background

Amino acids as the predominant transport form of nitrogen nutrition play important roles in nitrogen signals, metabolism and abiotic stress in most plants [1]. Therefore it is very important for plants to maintain amino acids homeostasis in cells by absorption and transport. Previous studies have demonstrated that the concentration of amino acids in cytoplasm, vacuole and even outside of cells is dynamically changing and intracellular and intercellular transport of amino acids is mediated by amino acid transporters within plant [2, 3]. Although acid transport systems are not highly specific for individual amino acids, transporters with some specificity for acidic or basic amino acids have been found in many species.

In plants there are almost 100 genes identified to encode putative amino acid transporters and majority of these genes are classified into the amino acid-polyaminecholine (APC) superfamily and the amino acid transporter (ATF1) superfamily [4]. Most characterized amino acid transporters from plants are belonging to the ATF1 superfamily and of them the amino acid permease (AAP) family is the most well studied subfamily [5–9]. The APC family of plants is divided into three subfamilies: amino acid/choline transporters (ACTs), cationic amino acid transporters (CATs) and polyamine H^+^-symporters (PHSs) [10, 11]. The CAT transporters play indispensable roles in maintaining metabolic functions, such as synthesis of nitric oxide (NO) and proteins, biosynthesis of polyamine, and flow of amino acids, by mediating the bidirectional transport of cationic amino acids in plant cells. The CAT transporters locating at the membrane of cell or vacuole contain between 11 and 14 putative transmembrane (TM) domains and they are basic amino acid transporters with high affinity [2, 12, 13].

A few genes of the *CAT* family have been studied in plants. The first cloned amino acid transporter of APC-type proteins in plant was AAT1, which was renamed to CAT1 to emphasize its structural and functional similarity with mammalian CATs [4, 12]. Because *CAT1* gene allows cells to resume growth when amino acids are available, so it is indispensable for cell survival during stress. In *Arabidopsis*, *AtCAT1* is expressed in leaves, flowers and developing siliques, and the gene product is localized at the plasma membrane [2, 12]. Functional identification of *AtCAT1* gene has been indicates that it mainly transports cationic amino acids and might play multiple roles in phloem development [12]. Overexpressing *CAT1* in *Arabidopsis* reduced the total biomass of transgenic plants and accelerated the flowering time, while the resistance to the hemibiotrophic bacterial pathogen *P. syringae* was enhanced through constitutively activating the salicylic acid (SA) pathway [14].

The *CAT* genes family in *Arabidopsis* is comprised by 9 genes. *AtCAT2* and *AtCAT4* were primarily localized at the tonoplast, while *AtCAT3* was localized to the endoplasmic reticulum and *AtCAT1*, *AtCAT5* and *AtCAT6* were localized at the plasma membrane [2, 14, 15]. The concentration of amino acids in *cat2* mutants was increased, which indicates that *AtCAT2* is essential for maintaining total tissue amino acid concentrations in plant cells [15]. *AtCAT5*, as a high-affinity, cationic amino acid transporter, may play roles in re-uptaking of leaked amino acids [2]. *AtCAT6*, which is expressed in lateral root primordia, flowers and seeds, transports large, neutral and cationic amino acids and plays a role in providing amino acids to these tissues of plants [13]. *AtCAT7* was not characterized because there was no EST or cDNA could be identified. Interestingly, *AtCAT8* is not only particularly localized on the tonoplast, but also identified on the plasma membrane and autophagosomes membranes [2, 16]. The expression level of *AtCAT8* is higher in young and rapidly dividing tissues, such as root apical meristem and young leaves. *AtCAT9* mainly locates in the vesicle membrane and participates in vacuolar transport. Overexpression of *AtCAT9* generally affects total soluble amino acid concentrations, slightly delays development, and ultimately improves survival rate under severe nitrogen starvation [17].

Besides in *Arabidopsis*, the *CAT* gene family has been identified and functionally investigated in poplar and rice [18, 19]. As the same described in *Arabidopsis*, twelve members of the poplar *CAT* gene family are characterized during leaf senescence and phylogenetically classified into four groups. Compared to other *CAT* genes, *PtCAT3*, *PtCAT4* and *PtCAT8* were not affected by leaf senescence. The expression of *PtCAT11* increased in senescing leaves and functionally characterized as a glutamine transporter, which indicates that *PtCAT11* may play a key role in N remobilization during senescence by facilitating glutamine loading into phloem vessels in poplar. There are 11 genes in rice *CAT* gene family with *OsCAT1* downregulated and *OsCAT6* upregulated by drought stress. *OsCAT4* and *OsCAT11* may have no function in rice, because their expressions in all organs are negligible.

Based on the role of CATs in transport of cationic amino acids, *CAT* genes could be good target for crop improvement and resistance to abiotic stress. However, little is known about their functions in response to abiotic stress in plants. Moreover, to our knowledge, the *CAT* gene family in cotton is still poorly understood. Here, we performed a genome-wide and comprehensive study of the *CAT* gene family in four species of cotton (*G. arboreum*, *G. raimondii*, *G. hirsutum* and *G. barbadense*). Based on the results of gene structures, phylogenetic relationships, gene chromosomal localization and *cis*-elements analysis, the characteristics of *CAT* gene family were investigated. Moreover, we also conducted gene interaction network of the GhCAT proteins and expression patterns of *GhCAT* genes under different abiotic stresses. Our results lay a foundation for further study on the regulation mechanism of *CAT* genes in cationic amino acids transporting and distribution during plant development and responses to abiotic stress.

## Results

### Identification of *CAT* genes

A total of 14, 14, 26 and 25 CAT members were identified in four cotton species, respectively (Table 1, Table S1, S2 and S3). The 79 putative *CAT* genes were renamed based on cotton species and chromosomal locations. The *Gossypium* CAT proteins showed conservative physical properties. Most CAT proteins are similar in amino acid (AA) lengths, molecular weights (MWs), and theoretical isoelectric points (pI). The GaCAT, GrCAT, GhCAT and GbCAT had 558–663, 568–644, 373–642, and 335–642 AA, respectively. The MWs of the CAT proteins varied from 36.222 kDa (GbCAT1A) to 71.238 kDa (GaCAT6) and the isoelectric points of the CAT proteins ranged from 5.776 (GhCAT14D) to 9.037 (GhCAT10D and GbCAT10D) (Table 1, Table S1, S2 and S3).

**Table 1:** Information of the *CAT* genes in *G. hirsutum*

Gene ID	Locus ID	Chromosome Position	Gene Length (bp)	Protein Length (aa)	Molecular Weight (kDa)	Isoelectric Point	Subcellular Prediction
GhCAT1A	GH_A03G2427.1	A03:111,413,132–111,415,485:-	2,354	596	64.936	7.116	PM
GhCAT2A	GH_A04G1635.1	A04:86,446,065–86,448,451:-	2,387	604	65.867	7.69	PM
GhCAT3A	GH_A08G1649.1	A08:105,024,365–105,027,559:-	3,195	568	60.757	7.751	PM
GhCAT4A	GH_A08G1890.1	A08:111,828,499–111,831,382:-	2,884	572	62.901	8.44	PM
GhCAT5A	GH_A10G2168.1	A10:106,629,101–106,631,753: +	2,653	586	63.673	8.185	PM
GhCAT6A	GH_A11G0270.1	A11:2,375,780–2,377,849: +	2,070	573	63.311	8.819	PM
GhCAT7A	GH_A11G2542.1	A11:91,300,183–91,305,351:-	5,169	638	67.488	6.422	PM
GhCAT8A	GH_A11G2543.1	A11:91,396,766–91,401,213:-	4,448	642	68.65	6.809	PM
GhCAT9A	GH_A12G2332.1	A12:101,658,525–101,661,185:-	2,661	574	63.035	8.439	PM
GhCAT10A	GH_A13G0099.1	A13:1,022,015–1,024,585:-	2,571	373	41.034	7.108	PM
GhCAT11A	GH_A13G1785.1	A13:97,979,306–97,981,030: +	1,725	574	62.696	8.876	PM
GhCAT12A	GH_A13G1894.1	A13:101,053,047–101,058,437:-	5,391	642	68.427	5.92	PM
GhCAT1D	GH_D01G1558.1	D01:38,670,727–38,672,505: +	1,779	592	64.57	8.627	PM
GhCAT2D	GH_D02G2589.1	D02:69,573,127–69,575,468:-	2,342	592	64.594	7.026	PM
GhCAT3D	GH_D04G1984.1	D04:55,428,621–55,430,990:-	2,370	606	65.963	6.97	PM
GhCAT4D	GH_D08G1661.1	D08:52,817,345–52,820,611:-	3,267	595	63.945	7.589	PM
GhCAT5D	GH_D08G1905.1	D08:57,570,390–57,573,276:-	2,887	572	62.79	8.442	PM
GhCAT6D	GH_D10G2269.1	D10:58,692,900–58,695,543: +	2,644	587	63.368	8.007	PM
GhCAT7D	GH_D11G0280.1	D11:2,282,854–2,284,925: +	2,072	573	63.218	8.819	PM
GhCAT8D	GH_D11G2594.1	D11:50,979,122–50,984,328:-	5,207	642	68.002	6.277	PM
GhCAT9D	GH_D11G2595.1	D11:51,055,576–51,060,015:-	4,440	642	68.884	7.216	PM
GhCAT10D	GH_D12G0071.1	D12:881,525–883,282: +	1,758	585	64.404	9.037	PM
GhCAT11D	GH_D12G2348.1	D12:55,947,670–55,950,313:-	2,644	572	62.843	8.671	PM
GhCAT12D	GH_D13G0103.1	D13:878,919–882,054:-	3,136	582	63.555	8.722	PM
GhCAT13D	GH_D13G1738.1	D13:53,274,172–53,275,896: +	1,725	574	62.715	8.58	PM
GhCAT14D	GH_D13G1844.1	D13:55,764,345–55,769,684:-	5,340	642	68.525	5.776	PM

*PM* Plasma membrane

### Phylogenetic analysis of the *CAT* family

**Fig. 1:**
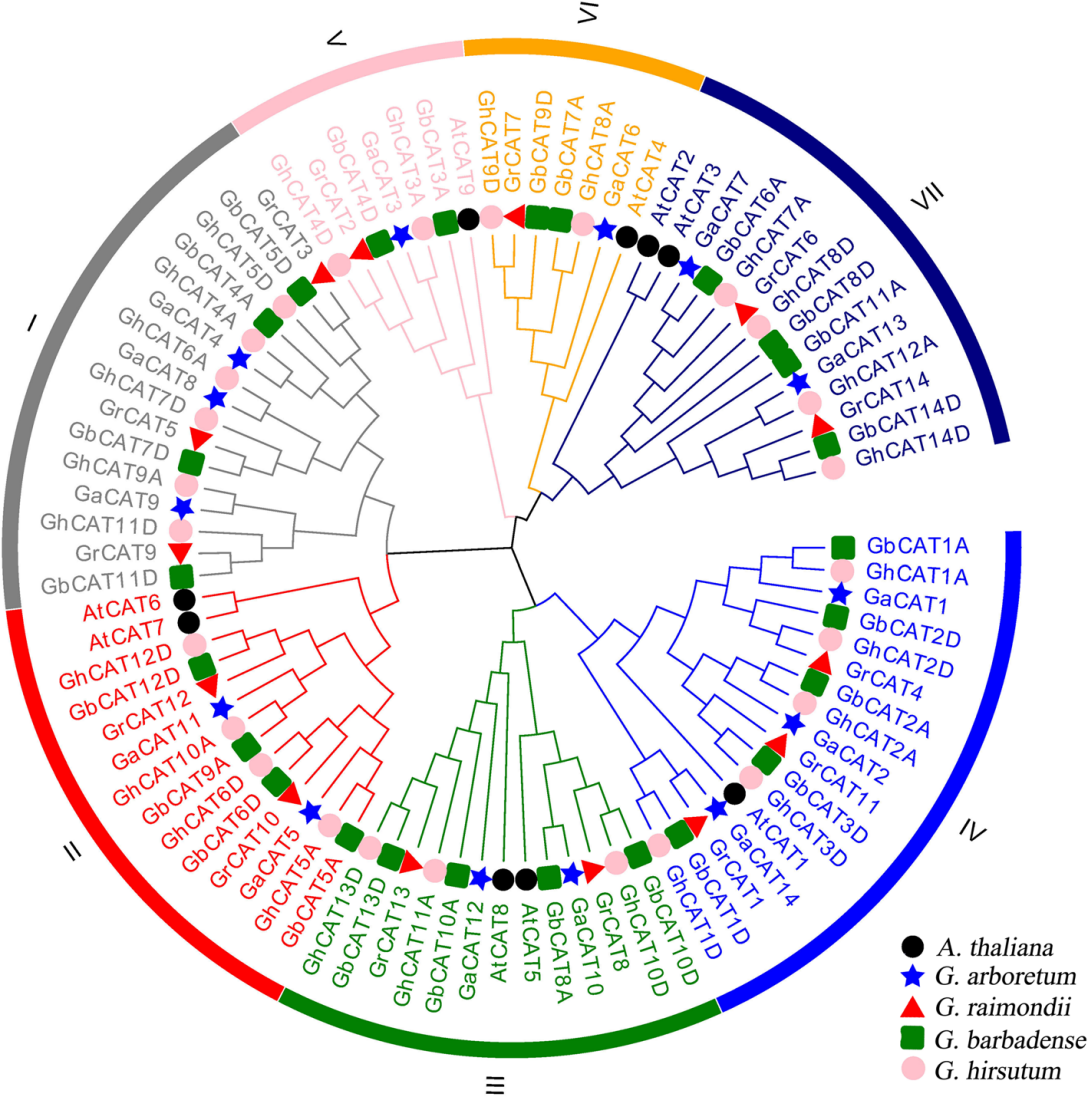
Phylogenetic relationship among CAT proteins. The neighbor-joining phylogenetic tree was constructed by MEGA 7.0 for the CAT proteins from *A. thaliana* (At), *G. arboreum* (Ga), *G. raimondii* (Gr), *G. hirsutum* (Gh) and *G. barbadense* (Gb)

To examine the evolutionary relationships of the *CAT* genes in cotton and *Arabidopsis thaliana*, we constructed a neighbor-joining phylogenetic tree using the full-length CAT proteins (Fig. 1). The CAT proteins were classified into 7 subgroups (I to VII), and each contained 16, 14, 17, 13, 7, 7 and 14 members, respectively. In terms of *Gossypium* CATs, the total number in *G. arboreum* and *G. raimondii*, was 28, which was nearly equal to that in *G. hirsutum* and *G. barbadense*. All *CAT* genes of *G. hirsutum* and *G. barbadense* were clustered together as either *G. raimondii* or *G. arboreum CAT* genes. This finding was consistent with the hypothetical origins and history of allotetraploid cotton.

### Structure of *CAT* genes and conserved motifs

**Fig. 2:**
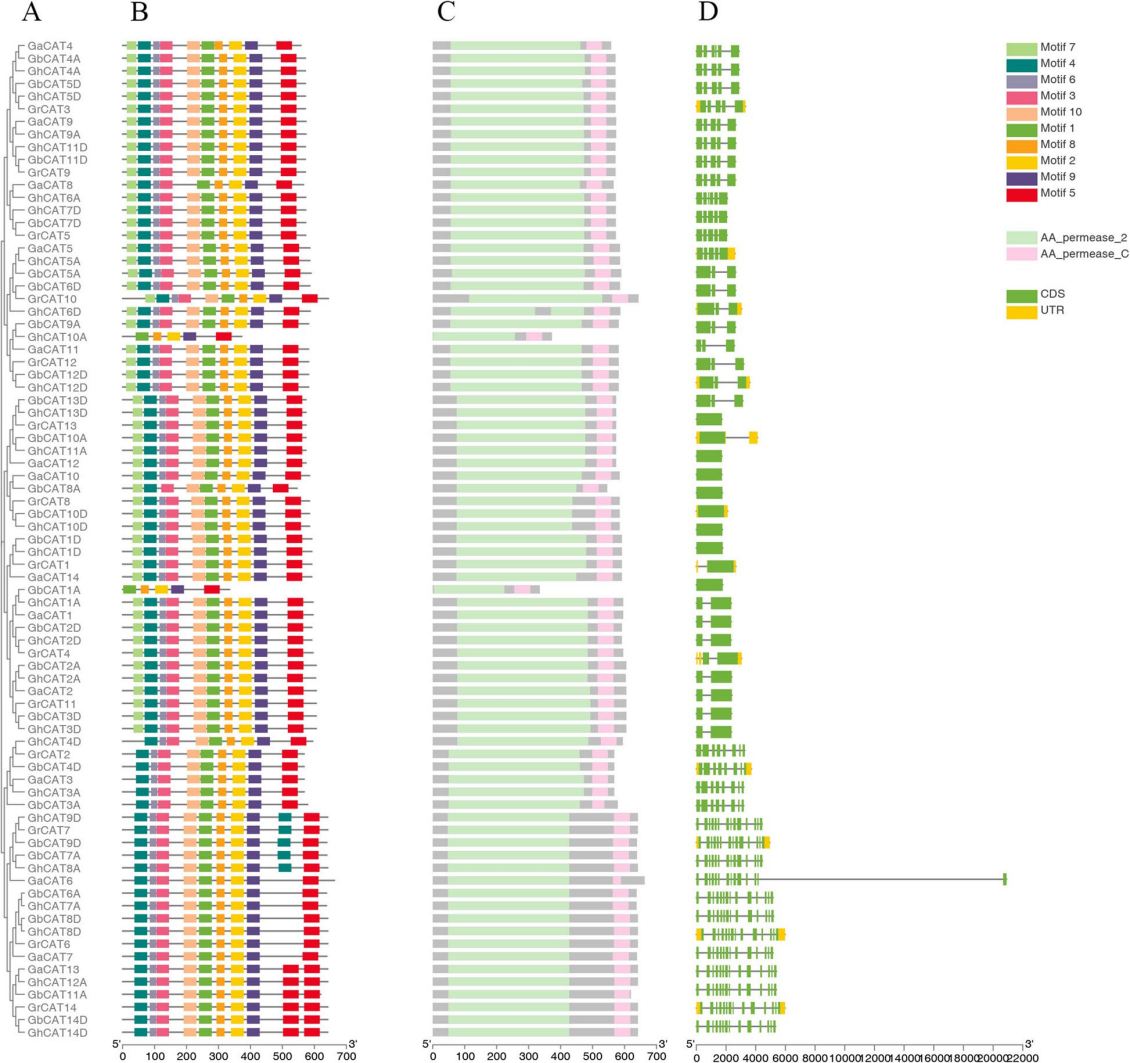
Structural analysis of *CAT* genes in *G. arboreum*, *G. raimondii*, *G. hirsutum* and *G. barbadense*. **A** Phylogenetic tree of CAT proteins from *G. arboreum*, *G. raimondii*, *G. hirsutum* and *G. barbadense*. **B-C** Motifs and domains (**C**) of *Gossypium CAT* proteins. **D** Structures of *Gossypium CAT* genes

The exons, introns, protein domain and conserved motifs of the *CAT* genes were analyzed (Fig. 2). The results showed that *CAT* genes from the same subgroup owned similar genetic structure and exon number. Ten specific motifs were defined and named motif 1 to 10. The CAT proteins showed similar conserved motif compositions and all CAT proteins contain motif 1, 2, 5, 8 and 9, suggesting that these five motifs are key components for CAT protein sequences. In a word, members of the same subgroup have similar gene structure and motif compositions, while genes of different subgroups possessed specific structure, suggesting that the *CAT* gene family was functionally conserved and diverse during evolution. *CAT* genes in subgroup I to VIII possess 5, 3, 1, 2, 8, 14 and 14 exons, respectively. However, *GbCAT8A* in subgroup III contains 3 exons and GbCAT1A in subgroup IV contains 1 exon. Two domains, AA_permease_2 and AA_permease_C, were highly conserved in all the CAT proteins.

### Chromosomal distribution and selection pressure analysis

**Fig. 3:**
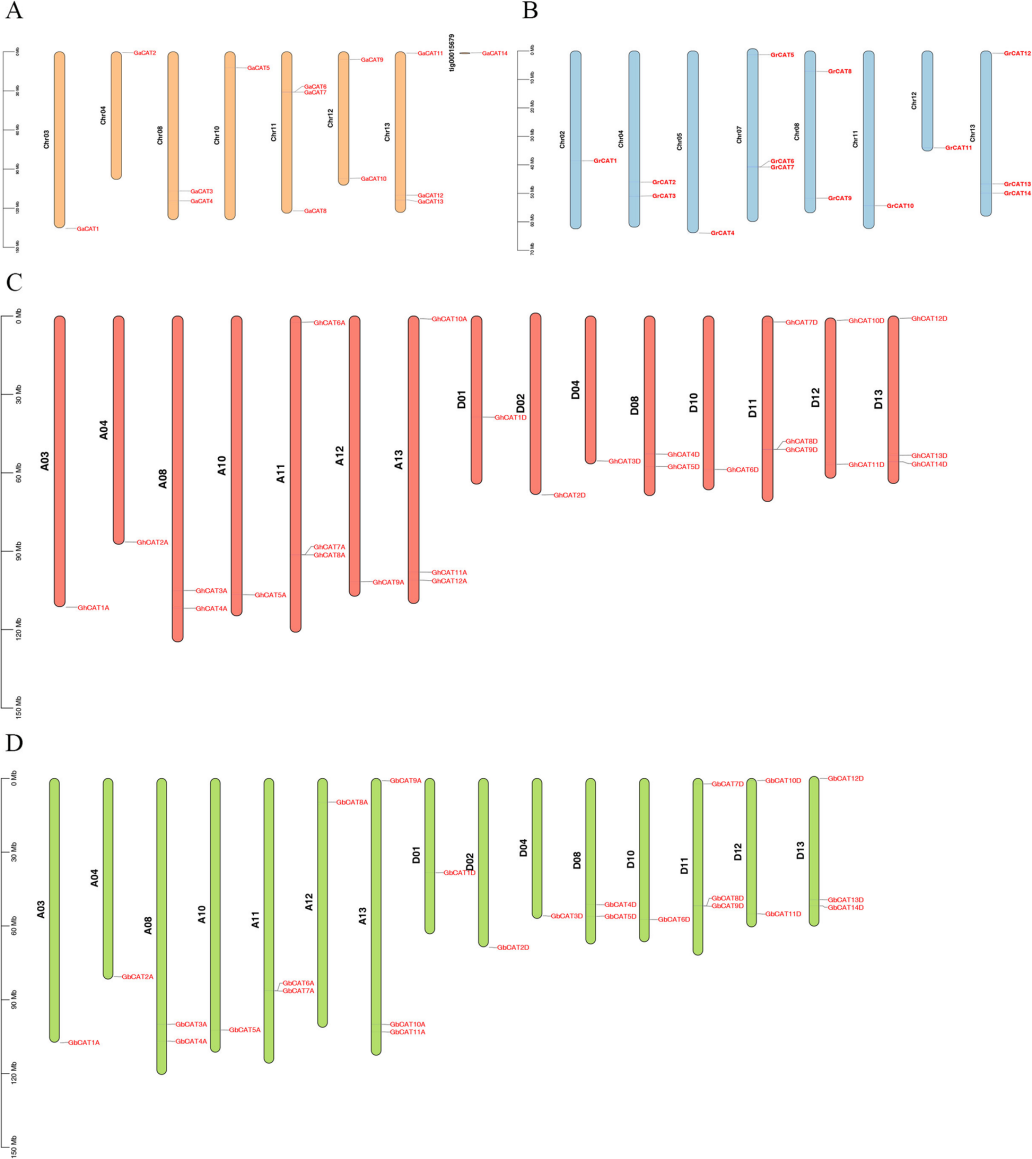
Chromosomal locations of *CAT* genes in *G. arboreum*, *G. raimondii*, and *G. hirsutum* and *G. barbadense*. **A-D** Chromosomal locations of *CAT* genes in *G. arboreum* (**A**), *G. raimondii* (**B**), *G. hirsutum* (**C**), and G. *barbadense* (**D**)

To further explore the relationship between the genetic divergences of the *CAT* gene family, all *CAT* genes were mapped to their corresponding chromosomes. Of the 79 cotton *CAT* genes, 78 were located on the chromosomes of four cotton species. For the *GaCAT* gene family, 13 out of 14 *CATs* were allotted up to 7 of the 13 *G. arboreum* chromosomes, and the remaining gene, *GaCAT14* showed affinity with yet unmapped scaffolds (Fig. 3). For the *GrCAT* gene family, all 14 CATs were allotted to 8 of the 13 *G. raimondii* chromosomes (Fig. 3). For the *GbCAT* and *GhCAT* gene families, they have the similar pattern of chromosomal distribution and all *CATs* were allotted to 15 of the 26 chromosomes, respectively (Fig. 3). However, there is a different of gene distribution on the GhAt12 and GbAt12 and one more gene *GhCAT6A* is mapped on the GhAt11.

**Fig. 4:**
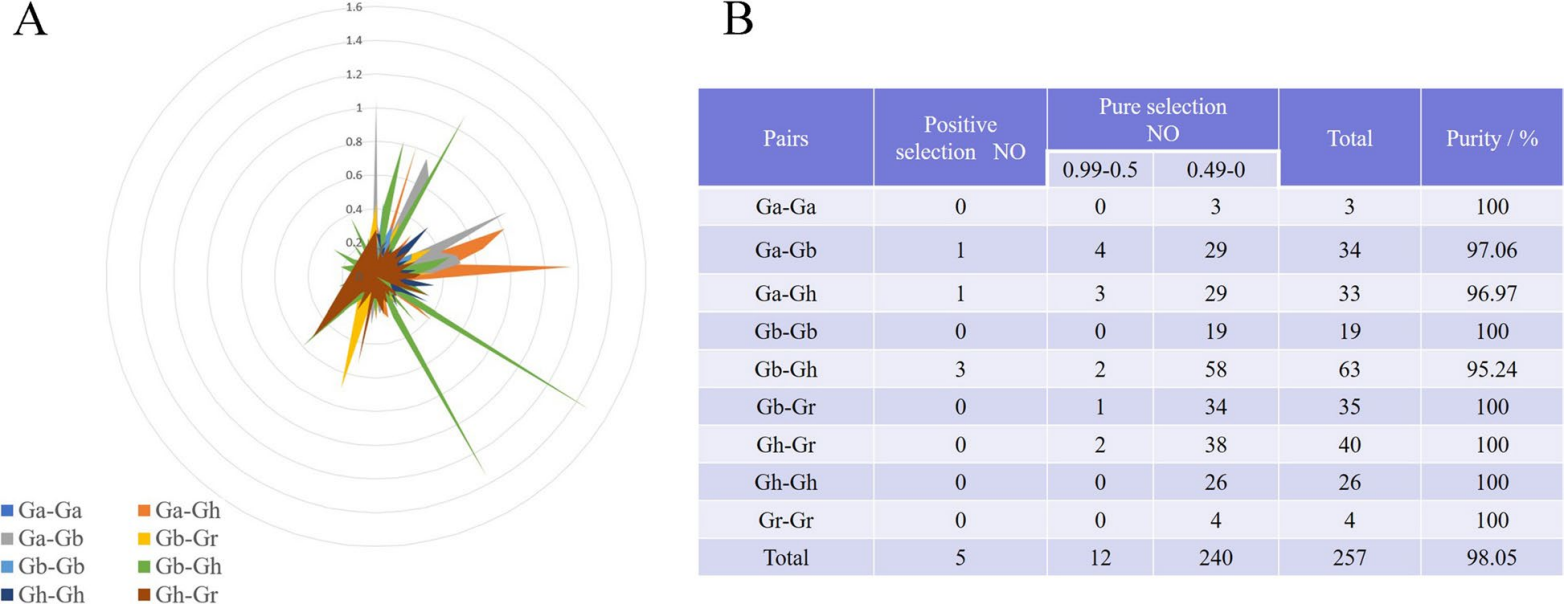
Analysis of non-synonymous (Ka) to synonymous (Ks) ratio. (**A**) nonsynonymous (Ka) and synonymous (Ks) divergence values for Ga–Ga, Ga-Gb, Ga-Gr, Ga-Gh, Gb-Gb, Gb-Gr, Gb-Gh, Gr-Gr, Gr-Gh and Gh-Gh are shown in circular chart. (**B**) Prediction number of duplicated gene pairs involved in different combinations from four cotton species

In the course of evolution, repeated gene pairs may lose original functions and acquire new functions. To study the selection pressure for the segmental duplication of *CAT* gene pairs, the ratio of Ka and Ks of the comparable parts was calculated (Fig. 4A, Table S4). The results indicated that the Ka/Ks ratios of most segmental duplications of *CAT* gene pairs were less than 1, suggesting that they had experienced purifying selection pressure after gene duplication events. Due to the constraints of purification selection on divergence, most segmental duplications of the CAT pairs might show similar functions. *GbCAT1A/GaCAT1*, *GhCAT10A/GaCAT11*, *GbCAT3A* /*GhCAT3A*, *GbCAT11A*/*GhCAT12A* and *GbCAT1D*/*GhCAT1D* presented a Ka/Ks ratio greater than 1, demonstrating that these *CAT* gene pairs had undergone positive selection during cotton evolution. The Ka/Ks value of Gb-Gr and Gh-Gr is equal to 1(Fig. 4B), indicating that the two cotton species are neutral selection. However, in Ga-Gb, Ga-Gh and Gb-Gh, there are 1 pair, 1 pair and 3 pairs with a Ka/Ks value greater than 1(Fig. 4B), which indicates that these genes have undergone positive selection during evolution.

### Analysis of promoters and differentially expressed genes

**Fig. 5:**
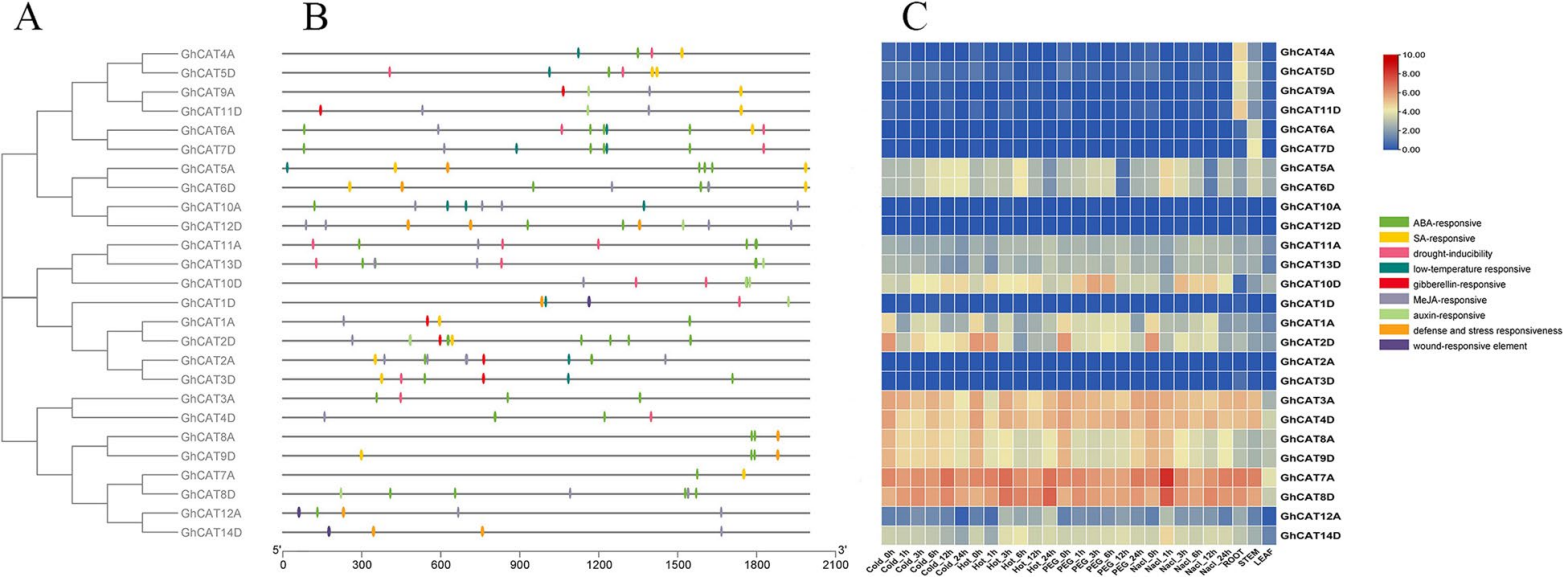
Analysis of promoters and differentially expression level of *GhCAT* family genes. **A** Phylogenetic tree of *GhCAT* genes. **B** *Cis*-elements in promoters of *GhCAT* genes. **C** Differentially expression level of *GhCAT* genes under cold, hot, salt and PEG stress and their expression level in root, stem and leaf

Many *cis*-acting elements were identified in the promoter region of each *GhCAT* gene by using PlantCARE (Fig. 5B), which could be classified into two types. The first important type is plant hormones responsive elements, which include five kinds of elements (abscisic acid, salicylic acid, gibberellin, jasmonic acid methyl ester and auxin responsive motifs). Abscisic acid (ABA) responsive element is the most widespread element related to the response to abiotic stress, and 21 *GhCAT* promoters contain this element. Seventeen *GhCAT* promoters contained jasmonic acid methyl ester (MeJA) responsive element and 7 promoters contained auxin (IAA) responsive element. The salicylic acid (SA) responsive element and the gibberellin (GA) responsive element were found in the promoter regions of 13 and 6 *GhCAT* genes, respectively. The other important type is abiotic stress responsive elements, which contains four kinds of elements (drought-inducibility, low-temperature responsive, defense and stress responsiveness and wound-responsive motifs). The drought-inducibility element and low-temperature element were located on the upstream of 10 and 8 *GhCAT* genes, respectively. Eight *GhCAT* promoters contained defense and stress responsiveness elements and wound-responsive elements were also observed in 3 *GhCAT* promoters. Moreover, we constructed the phylogenetic tree of *GhCAT* genes and found that the promoter regions of most homologous genes located on subgroups A and D contain the same cis-acting elements (Fig. 5A, B). These results suggest that *GhCATs* containing these *cis*-acting elements may play important roles in response to abiotic stress.

We analyzed the different expression levels of *GhCAT* gene family members in root, stem and leaf (Fig. 5C). We found that *GhCAT2D*, *GhCAT4A*, *GhCAT5D*, *GhCAT9A*, *GhCAT10D*, *GhCAT12A* and *GhCAT14D* genes had the highest expression levels in root, but some genes such as *GhCAT5A*, *GhCAT6D*, *GhCAT6A* and *GhCAT7D* genes had the highest expression levels in stem, only the *GhCAT10D* gene had the highest expression levels in leaf, while *GhCAT3A*, *GhCAT4D*, *GhCAT7A* and *GhCAT8D* were mainly expressed in root and stem. Interestingly, five genes including *GhCAT1D*, *GhCAT2A, GhCAT3D, GhCAT10A* and *GhCAT12D* did not express in three tissue. These results indicated the expression of *GhCAT* family genes was inconsistent in different tissues. Most *GhCAT* genes have obvious tissue specificity, which is largely related to their function in different tissues.

To further explor the responsive mechanism of *GhCAT* genes against abiotic stress, RNA-seq data of cotton leaves was used to analyze the differentially expression of genes under cold, heat, salt and PEG stress. The results suggested that the expression level of many genes varied under different stress (Fig. 5C), which showed that *GhCAT* genes participated in the regulation of abiotic stress, and the gene expression pattern from the same subfamily is similar (Fig. 5C). Under cold, heat, salt and drought treatments, 5 *GhCAT* genes were induced, including *GhCAT5A, GhCAT6D, GhCAT7A, GhCAT8D* and *GhCAT10D* and 6 *GhCAT* genes was repressed, such as *GhCAT1A, GhCAT2D, GhCAT8A, GhCAT9D, GhCAT11D and GhCAT14D*. However, the expression of some genes was not affected by these stresses, such as *GhCAT6A* and *GhCAT7D*. These results indicated that some *GhCAT* genes may play important roles in the regulation of abiotic stress.

### Expression analysis of *GhCAT* genes under drought and salt stress

**Fig. 6:**
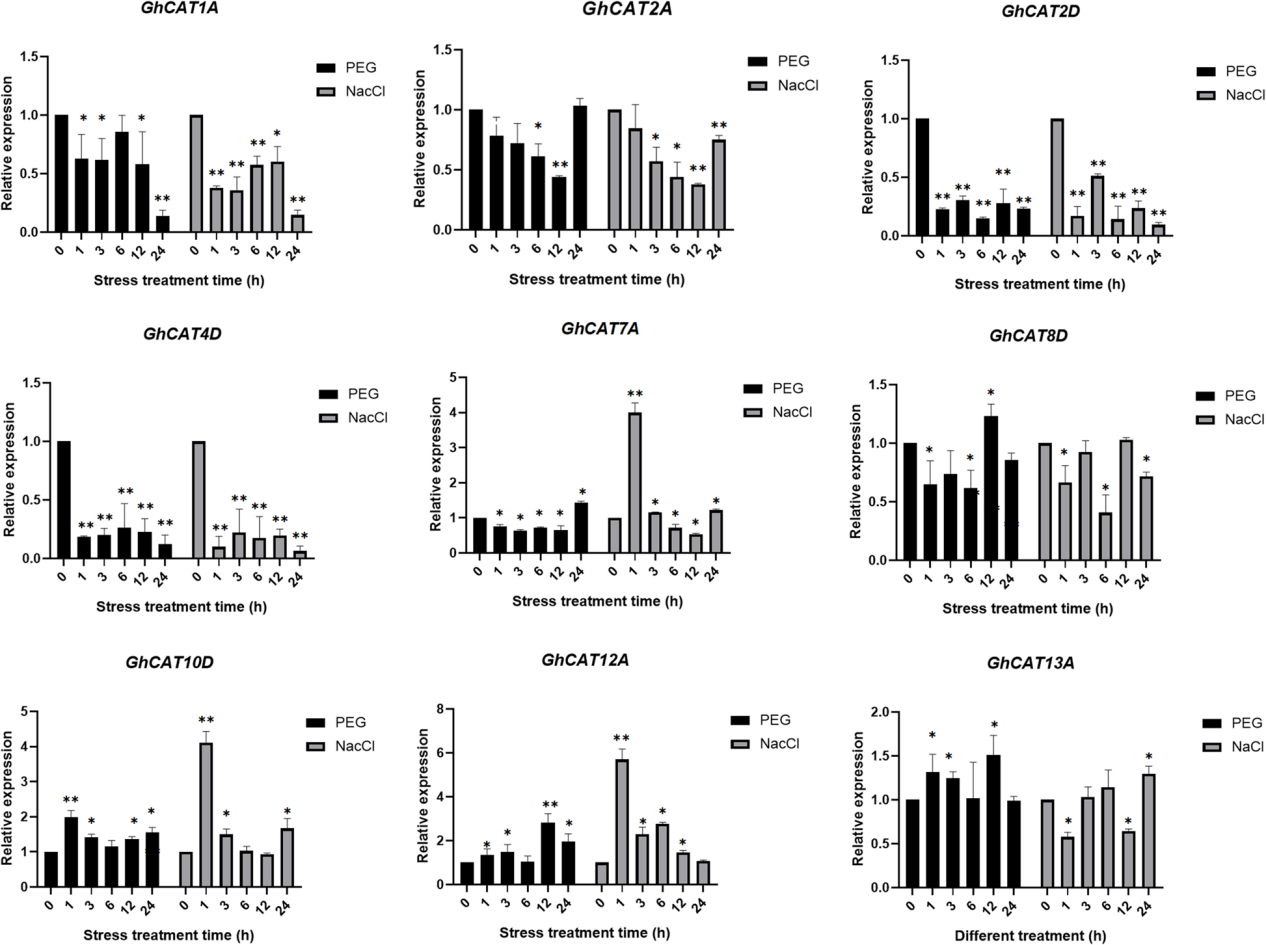
Validation of the expression patterns of *CAT* genes by qRT-PCR. Expression levels of the 9 *CAT* genes were normalized relative to that of the *GhHistone3* gene. Bars indicate the standard deviation of three technical replicates

To verify the expression profiles obtained from the transcriptome data for *GhCAT* genes under drought and salt stress, nine *GhCAT* genes were chose for qRT-PCR validation (Fig. 6). The results showed that some *GhCAT* genes could be induced by both treatments, such as *GhCAT10D*, *GhCAT12A* and *GhCAT13D*. The expression of *GhCAT7A*, *GhCAT10D* and *GhCAT12A* was rapidly up-regulated at 1 h after salt treatment, indicating that they might be involved in salt stress response. However, *GhCAT1A*, *GhCAT2A*, *GhCAT2D* and *GhCAT4D* were down-regulated under both stresses. The expression level of *GhCAT2D* and *GhCAT4D* decreased sharply after salt and drought stresses. Under both stresses, *GhCAT12A* gene was downregulated gradually with time (at 1, 3, 6 and 12 h) but was expressed normally at 24 h. In addition, the expression of *GhCAT8D* and *GhCAT13D* changed inordinately under either type of abiotic stress. These results suggest that *GhCAT* genes may play important roles in the regulation of drought and salt stresses response in *G. hirsutum*.

### Interaction network of GhCAT proteins

**Fig. 7:**
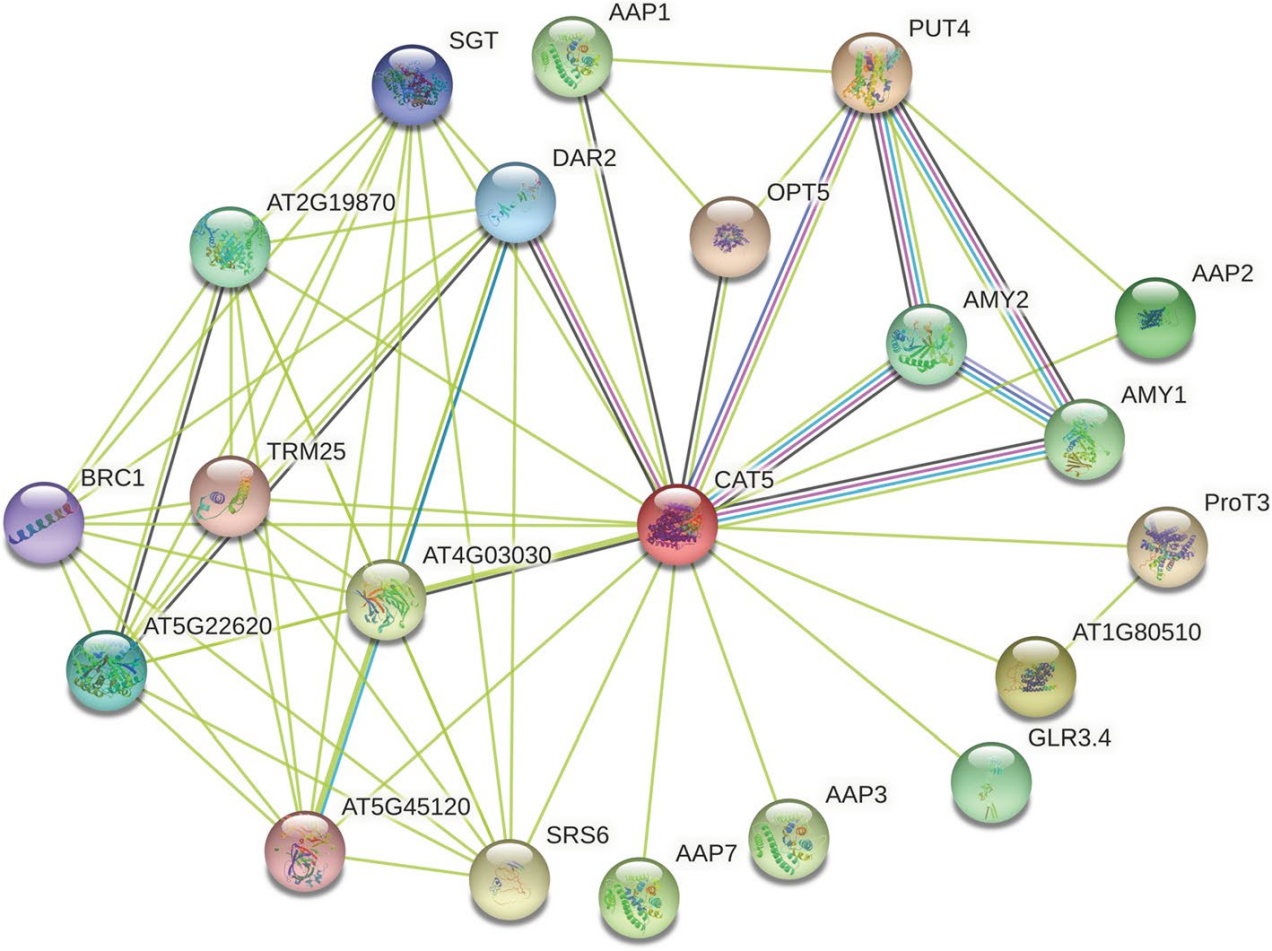
Interaction network of GhCAT protein

To further investigate the function of GhCAT protein, we compared GhCAT10D protein to *Arabidopsis thaliana*, and obtained the *Arabidopsis* homolog *AtCAT5* (AT2G34960.1), and predicted 20 proteins interacting with CAT (Fig. 7). Among these interacting proteins, AMY1 and AMY2 were identified to interact with GhCAT10D in Curated Databases. In human, AMY1 associated with obesity, plays important roles in impacting microbiome composition and function [20]. In *Arabidopsis thaliana* genome, AMY1 and AMY2 are α-amylases [21] and studies demonstrated the AMY1 in *Arabidopsis* leaves was secreted and induced by biotic and abiotic stress [22]. Among these interacting proteins, proteins associated with amino acid transportation are AAP1, AAP2, ProT3, AAP3 and AAP7. AAPs are amino acid permeases and AAP1 plays key roles in regulating the import of amino acid into root cells or developing embryo [23, 24]. We came to a conclusion that the *CAT* gene, together with AMY1 and AAP1, regulate the transport of cationic amino acid, thereby enhancing the defense mechanism against abiotic stress.

### Subcellular localization analysis of *GhCAT10D*

**Fig. 8:**
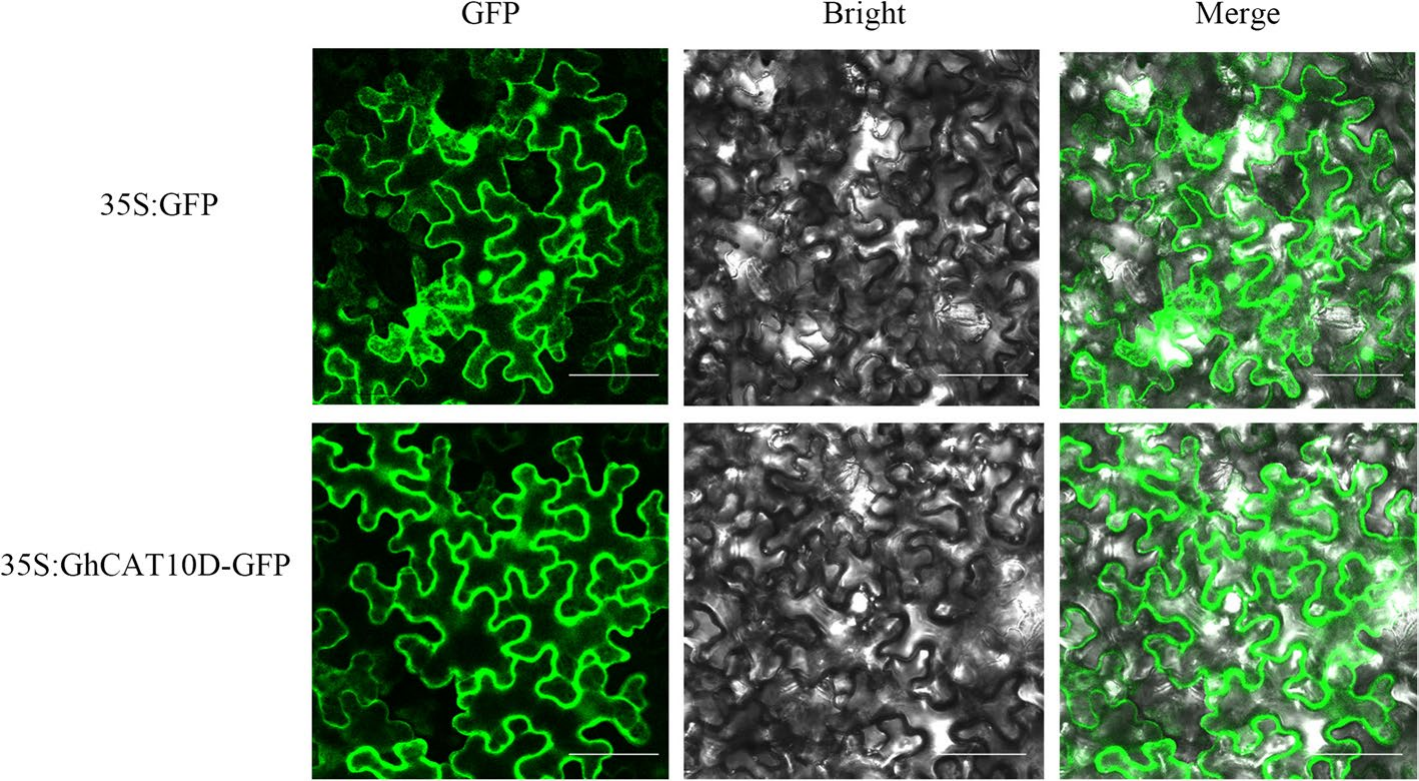
Subcellular localization of GhCAT10D in *N. benthamiana* epidermal cells. Agrobacterium-infiltrated *N. benthamiana* leaves expressing the GFP-GhCAT10D fusion protein driven by the 2 X CaMV35S promoter. Confocal images of the cells expressing GhCAT10D are showing their distribution on the plasma membrane. Cells transformed with vector only (CaMV35S-GFP) are shown on the top. Scale bar = 30 μm

All the CAT proteins of four cotton species were located on plasma membrane through bioinformatics analysis. To determine the site of residence, we performed subcellular localization analysis of *GhCAT10D* gene. For the transient expression assays, *GhCAT10D* gene was fused at the C terminus to the GFP reporter gene and the construct was expressed in transformed *N. benthamiana* epidermal cells. Confocal microscopy showed that GFP fluorescence of GhCAT10D was localized mainly at the periphery of the cell. Therefore GhCAT10D was probably localized on the plasma membrane (Fig. 8).

### *GhCAT10D* gene plays a key role in salt tolerance of cotton

**Fig. 9:**
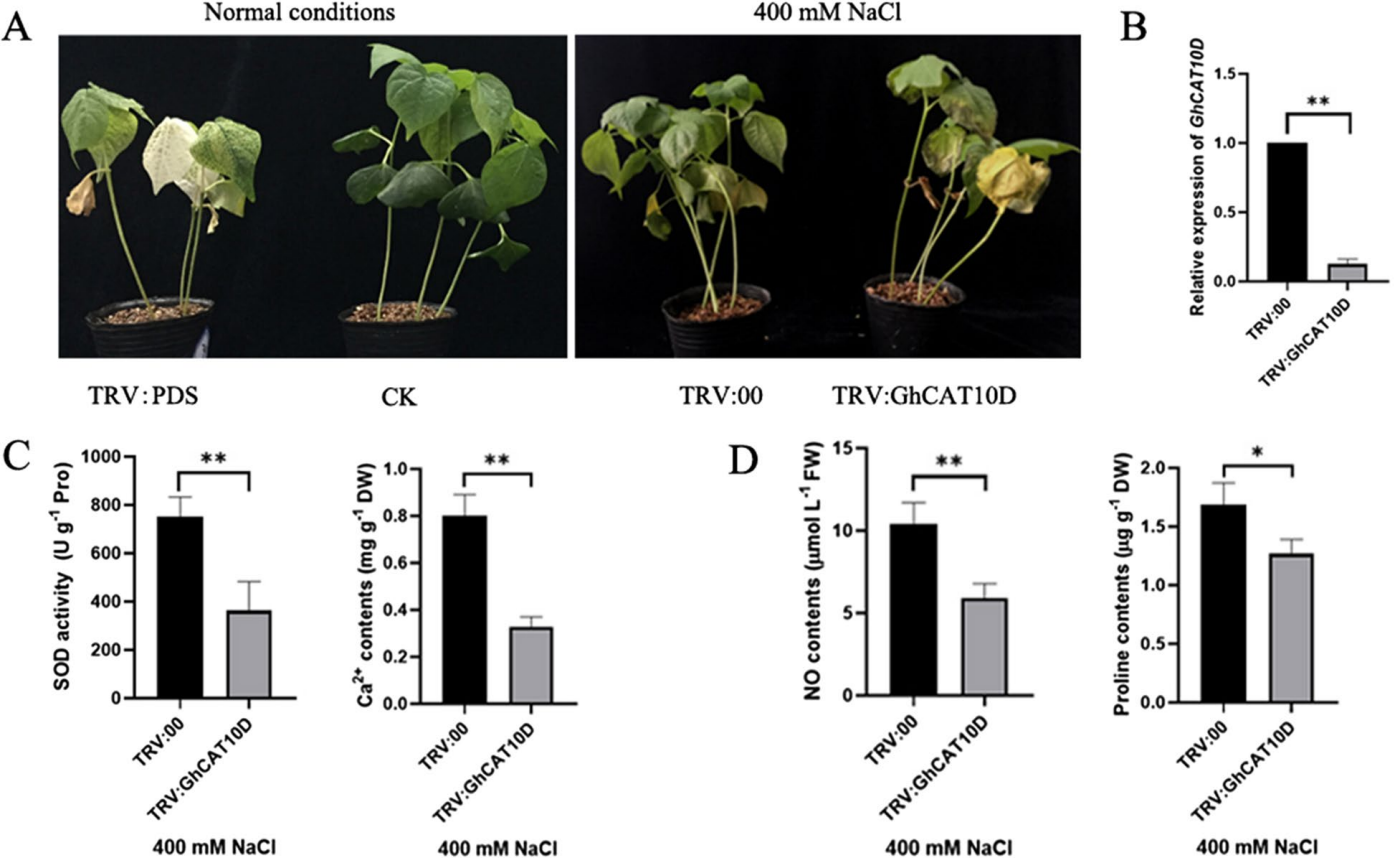
Function verification of *GhCAT10D*. **A** Phenotypic comparison of *GhCAT10D* silenced plant under salt stress. **B** Detection of *GhCAT10D* silencing efficiency. **C** Detection of SOD activity and Ca^2+^ content. **D** Detection of NO and proline content. *p < 0.05, **p < 0.01

A VIGS experiment was performed to verify the potential roles of *GhCAT10D* in cotton response to salt stress. After VIGS, the level of *GhCAT10D* expression in leaves of the *TRV:GhCAT10D* plants dramatically decreased compared with the *TRV:00* plants (Fig. 9A), indicating the strong and specific silencing of *GhCAT10D*. And after 400 mM NaCl treatment, compared with the *TRV:00* plants, the *TRV:GhCAT10D* plants were more sensitive to salt stress, implying this gene contributes to the salt tolerance of cotton (Fig. 9B). In order to further determine the function of *GhCAT10D* gene in the regulation of salt tolerance in cotton, SOD activity and Ca^2+^ content were measured in the leaves of *TRV:00* and *TRV:GhCAT10D* plants (Fig. 9C). The content of ROS in the *TRV:GhCAT10D* plants was significantly higher than the *TRV:00* plants, while the amount of Ca^2+^ in *TRV:GhCAT10D* plants was markedly down-regulated by gene silencing.

As the CATs play key roles in transporting of cationic amino acids, so we want to know the changes in the amount of the metabolites associated with cationic amino acids in the cells under salt stress. The contents of nitric oxide (NO) and proline were determined in the *TRV:00* and *TRV:GhCAT10D* plants after salt treatment (Fig. 9D). Compared with the *TRV:00* plants, the contents of NO and proline were significantly decreased in the TRV:GhCAT10D plants.

## Discussion

Being an important cash crop, upland cotton is cultivated worldwide and facing severe biotic and abiotic stresses. The cationic amino acid transporters (CATs) play important roles in various biological process including plant growth and development along with resistance to abiotic stresses. Several *CATs* have been identified in *Arabidopsis thaliana* [2, 15], *Solanum lycopersicum* [25, 26], *petunia hybrid* [27, 28], *Populus tremula* [18] and *Oryza sativa* [19] previously. However, cotton was still lacking any type of studies about *CATs*. In our study, we performed a complete identification of *CAT* genes in *G. arboreum*, *G. raimondii*, *G. hirsutum* and *G. barbadense*, with the aim of understanding the roles of this gene family in cotton.

The CAT proteins are predicted to have 11–14 transmembrane domains (TMs) and intracellular N-and C-termini [29] and theoretically they should be located on the plasma membrane. There are 9 genes in *Arabidopsis CAT* family. *AtCAT1*, *AtCAT5* and *AtCAT6* were localized on the plasma membrane, while *AtCAT2* and *AtCAT4* were primarily localized at the tonoplast and *AtCAT3* was localized to the endoplasmic reticulum [2, 14, 15]. *AtCAT8* is not particularly localized to the tonoplast, but also identified on the plasma membrane and autophagosomes membranes [2, 16]. *AtCAT9* is identified as mainly localized to vesicular membranes. In our study, we predicted that all *CATs* were localized on the plasma membrane and subcellular localization experiments suggested that *GhCAT10D* was localized on the cytomembrane, which is consistent with *AtCAT5*. As *CATs* play important roles in transporting of cationic amino acid across plasma membrane, the localization of more *GhCATs* should be studied in the future.

In the *GhCAT* promoter regions, several stress-response elements were identified, such as low-temperature responsive, drought-inducibility, defense and stress responsiveness and wound-responsive motifs, which indicated that *GhCAT* genes may play key roles in response to abiotic stress (Fig. 5). And also several phytohormone regulatory elements were found in the *GhCAT* promoters, which suggested that *GhCAT* genes probably participate in phytohormone signaling pathways. It has been reported that ABA accumulates under stress and plays a key role in the stress response and tolerance of plants, possibly coordinating the ROS signaling route [30]. In our study, the *cis*-acting element, ABA-responsive element (ABRE), was observed in 21 *GhCAT* genes promoter region (Fig. 5), which indicated that *GhCATs* may play a part in regulation of abiotic stress tolerance.

As we know that amino acid transport is notably regulated by abiotic stress, such as low temperature, salt and drought [31]. The expression of *AtCAT1* and *GhCAT6* were down-regulated by salt stress in *Arabidopsis* shoot, while *AtCAT6* were found to be markedly induced by salt and cold stresses in root [19]. Similarly, in this study, 5 genes (e.g. *GhCAT7A*, *GhCAT10D* and *GhCAT12A*) and 6 genes (e.g. *GhCAT1A*, *GhCAT2A*, *GhCAT4D*) in *CAT* gene family were evidently down- and upregulated under salt and drought stresses, respectively, (Fig. 6). These results suggests that the *GhCAT* genes may play a critical role in response to abiotic stress in cotton.

In our study, we found there were some AAPs interacted with GhCATs, such as AtAAP1, AtAAP2, AtAAP3 and AtAAP7. Previous studies indicated that plant amino acid transporters (AATs) family includes two main families: the amino acid/auxin permease (AAAP) superfamily and the APC superfamily [32]. AAP family belongs to AAAP superfamily and CAT family is a subfamily of APC superfamily [10]. *AtAAP1* was notably expressed in the endosperm and cotyledons and regulated the import of amino acid into root cells or developing embryo [23, 24]. The results of qRT-PCR validation showed that *GhCAT10D* was highly expressed at 1 h after drought and salt treatment (Fig. 7). So we believed that plant cells transport amino acids through AAPs and CATs to maintain cell growth and development and abiotic stress resistance. The molecular mechanism of *CATs* in regulating cationic amino acid transport under abiotic stress remains to be further studied.

**Fig. 10:**
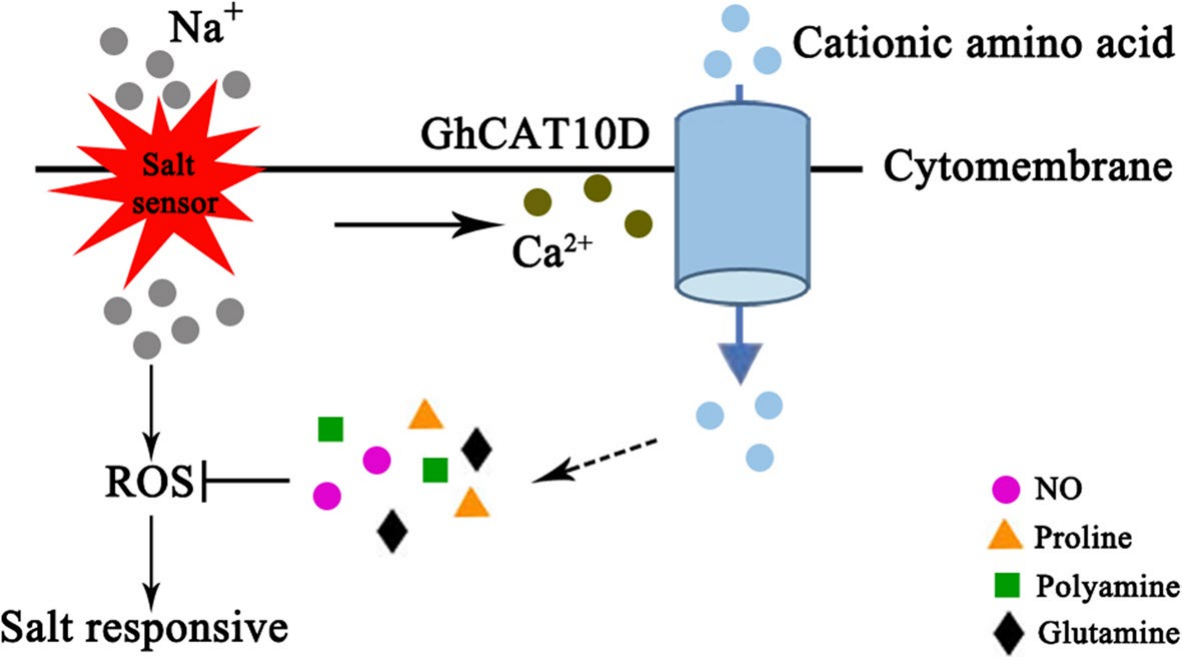
A proposed model illustrating the role of GhCAT10D in cotton providing salinity stress tolerance

Amino acids are well known as compositions of proteins, and their important roles in plant abiotic stress tolerance is often overlooked. Amino acid metabolism is closely related to carbohydrate metabolism, ammonium, protein synthesis and secondary metabolism. Amino acid transport proteins, such as CATs, facilitate the controlled exchange of amino acids across biological membranes. Amino acids being synthesized by different pathways are thus metabolized in different subcellular compartments [33]. During salt stress condition, cationic amino acid transporter GhCAT10D is activated by Ca^2+^ and takes cationic amino acids into cells. Cationic amino acids are raw materials for protein synthesis and other enzymatic reactions dependent on these amino acids, including the synthesis of NO, polyamines, proline and glutamine. These medium molecule substances scavenge ROS through specific molecular mechanisms [34] and provide salinity stress tolerance in cotton (Fig. 10).

## Conclusion

In this study, based on the results of gene structures, phylogenetic relationships, gene chromosomal localization and *cis*-elements analysis, the characteristics of *CAT* gene family were investigated. Moreover, we also conducted gene interaction network of the GhCAT proteins and expression patterns of *GhCAT* genes under different abiotic stresses. Our results lay a foundation for further research on the regulation of *CAT* genes in cationic amino acids transporting and distribution during plant development and responses to abiotic stress.

## Methods

### Identification of *CAT* family genes in cotton

In order to identify members of cotton *CATs*, we download the amino acid sequence of *Arabidopsis* *CATs* from the Arabidopsis Information Resource online databases (TAIR 10.1) (https://www.arabidopsis.org/). The four cotton genome files *Gossypium arboreum* (CRI, version 1.0), *G. raimondii* (JGI, version 2.0), *G. hirsutum* (ZJU, version 1.0) and *G. barbadense* (ZJU, version 1.0) were downloaded from the Cotton Functional Genomics Database (CottonFGD) (https://cottonfgd.net/). The amino acid sequences of *Arabidopsis Thaliana* *CATs* were used as queries to search against the genome sequences of four cotton species using local software Blast 2.13. Protein sequences of cotton CATs were submitted to ExPASy (http://web.expasy.org/protparam/) to predict the molecular weights (MW) and theoretical isoelectric points (pI) and charge.

### Phylogenetic analysis and classification of *CAT* genes

For phylogenetic analysis, all *CAT* amino sequences from *Arabidopsis thaliana* and four cotton species were aligned by ClustalX v1.83 with default parameters [35]. We used MEGA 7.0 to find best model and construct the neighbor-joining phylogenetic tree. The protein sequences of CATs identified in upland cotton were input into MEGA 7.0 software. ClustalW was used for multiple sequence alignment and the neighbor method was used to construct the intra species evolutionary tree. The specific parameters were as follows: Bootstrap Replication: 1000, Model/Method: P-distance, and all /Missing Data Treatment: Partial deletion.

### Calculation of Ka/Ks

The CDS sequences of *CAT* genes in the four cotton species were downloaded from CottonFGD. The homologous gene pairs of four cotton species were obtained by using the TBtools. We calculated the non-synonymous (Ka) and synonymous (Ks) substitution rates and Ka/Ks ratios by using the Kaks_Calculator 2.0 program [36], respectively.

### Analysis of expression patterns and cis-elements of *GhCAT* genes

For analyzing the expression profile of *GhCAT* genes under abiotic stress, the expression data was obtained from CottonFGD to analyze the expression level (fragments per kilo base of exon per million mapped, FPKM) of *GhCAT* family genes under cold, heat, salt and PEG stress. The 2000 bp DNA sequences in upstream of *GhCAT* genes were obtained from CottonFGD as promoters. We used the PlantCARE website (http://bioinformatics.psb.ugent.be/webtools/plantcare/html/) to predict *cis*-elements, which related to drought, plant hormones and other abiotic stress were kept for further analysis.

### Drought and salt stress treatment and qRT-PCR analysis

In order to investigate the expression pattern of *GhCAT* genes under drought and salt stress, nine genes (including *GhCAT10D*) were randomly selected from the *GhCAT* family. The seeds of upland cotton (zhong 9807) were cultivated on the sand medium and grew at 25℃ for 16 h in the day and 8 h at night in an indoor incubator. Under drought (12% PEG6000) and salt (400 mM NaCl) stress, the leaves were collected at 0, 1, 3, 6, 12 and 24 h for RNA extraction. The EASYspin Plus Plant RNA Kit was used to extract total RNA. The TransStart® Top Green qPCR SuperMix (+ DyeII) was used to reverse transcription of the extracted RNA to synthesize cDNA. Cotton *GhHistone3* (GenBank accession number: AF024716) was used as an internal control. The primers sequences used in qRT-PCR were shown in Table S5.

### Interaction network of *GhCAT* proteins

To investigate the interaction network of GhCAT protein, we obtain homologous genes by comparing the GhCAT10D protein sequences to *Arabidopsis thaliana*. The interaction network and function analysis of GhCAT protein was performed through STRING database (https://string-db.org/).

### Subcellular localization analysis

The *GhCAT10D* gene was constructed into pBI121 vector and *Agrobacterium tumefaciens* (GV3101) containing this vector or positive control (pBI121 vector with green fluorescent protein tag) was used to transfect the 6 week old *N. benthamiana* plants. The subcellular location of GhCAT10D-GFP proteins was analyzed after two days. Leaves of the transiently transformed *N. benthamiana* plants were used to visualize and localize the GFP protein under confocal laser-scanning microscopy (LSM 710, Carl Zeiss, Jena, Germany).

### Virus-induced gene silencing (VIGS) and salt treatment

A 300 bp fragment of *GhCAT10D* was inserted into pYL156 vector (which is cut with restriction enzyme XbaI and SrfI). The primers used are listed in Table S6. The pYL156 vector (*TRV:00)* and pYL156 vector containing *PDS* gene fragment (*TRV:PDS)* were used as the negative and positive controls, respectively. The recombinant plasmid (*TRV:GhCAT10D*) was transformed into *Agrobacterium* LBA4404. The procedure of infection was performed according to the method we reported previously [37]. The *TRV:00* and *TRV:GhCAT10D* plants were treated with water and 400 mM NaCl and photographed after 2 days. We then collected the second true leaves of plants to analyze the relative expression level of *GhCAT10D* gene.

### Detection of Ca^2+^, NO and proline content and superoxide dismutase (SOD) activity

The determination of Ca^2+^ content was performed according to the method we reported previously [38]. The detection of NO and proline contents and SOD activity were performed by using the nitric oxide (NO) assay kit, proline assay kit and superoxide dismutase (SOD) activity assay kit (Nanjing Jiancheng Bioengineering Institute, Nanjing, China), respectively.

### Statistical analysis

The GraphPad Prism 8.0 software was employed to analysis (ANOVA) the results. Duncan’s Multiple Range Test was used to compare the least significant difference of means (*p < 0.05, **p < 0.01).

## Supplementary Information

**Additional file 1: Table S1.** Information of the CAT genes in G. arboreum. **Table S2.** Information of the CAT genes in G. raimondii. **Table S3.** Information of the CAT genes in G. barbadense. **Table S4.** Analysis of Ka/Ks ratios of CAT genes in four cotton species. **Table S5.** Primers sequences used in qRT-PCR. **Table S6**. Primers sequences used in virus-induced gene silencing experiment.

## Data Availability

The datasets supporting the conclusions of the present study are included within this article and additional file. The *Arabidopsis* CAT protein sequences were downloaded from the Arabidopsis information source (TAIR) database (http://www.arabidopsis.org). The CAT nucleotide sequences and protein sequences of four cotton species and the RNA-seq data for expression profiles of *GhCATs* were downloaded from the CottonFGD (https://cottonfgd.net/).

## Abbreviations

CAT: Cationic amino acid transporters
NO: Nitric oxide
APC: Amino acid-polyaminecholine
AAP: Amino acid permease
ACT: Amino acid/choline transporters
PHSs: Polyamine H^+^-symporters
MW: Molecular weights
pI: Isoelectric points
VIGS: Virus-induced gene silencing
SOD: Superoxide dismutase
ABA: Abscisic acid
MeJA: Jasmonic acid methyl ester
SA: Salicylic acid
GA: Gibberellin
